# Using an epidemiological model to explore the interplay between sharing and advertising in viral videos

**DOI:** 10.1038/s41598-024-61814-9

**Published:** 2024-05-18

**Authors:** Yifei Li, Li Shao

**Affiliations:** 1 School of Mathematics, Harbin Institute of Technology, Harbin, 150001 China; 2 School of Social Sciences, Harbin Institute of Technology, Harbin, 150001 China

**Keywords:** Applied mathematics, Information technology

## Abstract

How to exploit social networks to make internet content spread rapidly and consistently is an interesting question in marketing management. Although epidemic models have been employed to comprehend the spread dynamics of internet content, such as viral videos, the effects of advertising and individual sharing on information dissemination are difficult to distinguish. This gap forbids us to evaluate the efficiency of marketing strategies. In this paper, we modify a classic mean-field SIR (susceptible–infected–recovered) model, incorporating the influences of sharing and advertising in viral videos. We mathematically analyze the global stability of the system and propose an agent-based modeling approach to evaluate the efficiency of sharing and advertising. We further provide a case study of music videos on YouTube to show the validity of our model.

## Introduction

Viral videos, characterized by their rapid and expansive dissemination on social networks, have become a cultural phenomenon with profound implications for societal dynamics such as political campaigns and online marketing. For example, the video “Baby Shark Dance” on YouTube has over 11 billion views since 2016^1^. The phenomenon of viral videos has caused the shift of budgets of companies from mass media to online marketing activities^2^. Understanding how information goes viral has attracted attention in fields of psychology^3^, marketing^4,5^ and public health management^6^. Insights of the spread dynamics of viral videos would be useful to many parties such as political campaigners and product marketing managers.

Many studies employ epidemilogical models to comprehend the dynamics of information spreading^7–11^. Discussions of the analogy between infectious disease spreading and information dissemination can be traced back to Goffman and Newill^12^. Based on the classic SIR compartmental model^13^, epidemiological models have been applied in many areas of information dissemination such as rumor propagation^14^, viral marketing strategies^15^ and computer viruses propagation^16,17^. Similar to the spread of viruses in an epidemic due to the interactions between susceptible and infected individuals, information dissemination on the internet is primarily driven by the communications of online individuals. Epidemiological models are shown to generate matching results with the spread dynamics of various internet content. Bauckhage^18^ found that SIR (susceptible-infected-recovered) models give a good account of the dynamics of memes. Anand et al.^19^ reported that the data obtained from SI (susceptible and infected) models validated well on the view counts of YouTube videos. Sachak-Patwa et al.^20,21^ considered SEIRS (susceptible-exposed-infected-recovered- susceptible) models with time delay to precisely describe the change of view counts and long-term dynamics of music videos on YouTube. Agent-based models or network-based models are often applied to incorporate the heterogeneous structure of social networks^15,22,23^. Through a systematic application of natural language processing and hierarchical clustering algorithms, Ghosh et al.^24^ investigated a huge amount of survey data to provide deep insights into the understanding of quantitative modeling.

Existing models mainly focus on individual interactions on the internet and neglect the role of advertising^25^. Unlike the advertising strategy in viral marketing which encourages consumers to share product information with others, the advertising strategies for online videos are more abundant. For instance, in the case of social media influencer marketing, companies pay for content shearers who have a large number of followers to advertise their product^26^. This motivates us to embed a novel advertising mechanism into epidemiological models, which could strategically transform viewers into active sharers. It is expected that advertising acts as a catalyst, influencing the initial exposure of a video to potential viewers and amplifying its reach^2^. As advertising does not only trigger the information epidemic on the internet but also keeps affecting the spreading process, the interplay between person-to-person transmission and advertising needs to be further formalized so that mathematical tools can be used to provide more insights into the dynamics of information dissemination. Once the mathematical model is established, a methodology for using simulation algorithms and real data to validate the model and further evaluate advertising efficiency is crucial to qualitatively and quantitatively understand the spreading process.

In this work, we use a mean-field SIR model that incorporates the effect of advertising into the traditional modeling framework to explore the relationship between sharing and advertising. Since differential equation models can only reflect the population-level dynamics, we further propose an agent-based model, which is a stochastic discrete model related to a continuum description in the limiting case^27,28^, to simulate the dynamics of individual behaviors and connect the macroscopic and microscopic landscapes. The averaged data obtained from stochastic simulations would be consistent with the solutions of our continuous model and further provide the details of the spreading process. In particular, through labeling individuals with their paths of infection, we can quantitatively evaluate the efficiency of advertising.

The content of this work is organized as follows. In “The model” section, we formulate a susceptible-infected-recovered epidemic model, which divides the target people for marketing into three classes. A key feature of our model is that we use an *advertising function* associated with the number of active sharers to reflect the effect of advertising. In “Dynamics of the model” section, we analyze the global stability of our model and show how the interplay between sharing and advertising determines the size of populations in different classes. To further evaluate the effectiveness of advertising, in “Evaluate advertising efficiency using agent-based algorithm” section, we propose an agent-based modeling approach to measure the number of individuals who get their information through promotions from other individuals or advertisements. A case study of music videos on YouTube is presented in “Model validation against data” section to show the validation of our model. Finally, in “Discussion” section, we summarize our results and discuss the useful findings.

## The model

We compartmentalize the target people into three classes:

- *Susceptible* Potential audience who are not aware of the video.
- *Infected* Individuals who have watched the video and become active in sharing the video.
- *Recovered* Individuals who have watched the video and stopped sharing it. Once recovered, they will no longer be interested in watching or sharing.

**Figure 1:**
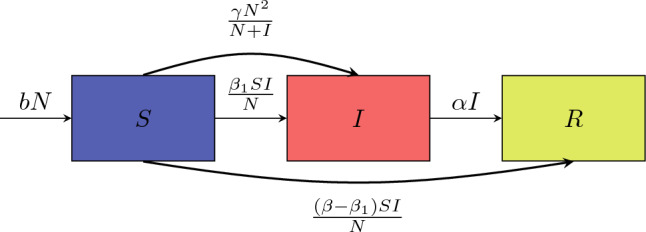
Diagram of the epidemic model.

The dynamics of individuals in these classes is described by an ordinary differential equations (ODEs) system

1 $$\begin{aligned} \left\{ \begin{aligned}{}&S'=-\frac{\beta IS}{N}-\frac{\gamma N^2}{I+N}+bN,\\&I'=\frac{\beta _1 IS}{N}+\frac{\gamma N^2}{I+N}-\alpha I,\\&R'=\frac{(\beta -\beta _1)IS}{N}+\alpha I, \end{aligned} \right. \end{aligned}$$

where *S*(*t*), *I*(*t*) and *R*(*t*) represent the number of susceptible, infected and recovered individuals, respectively, see Fig. 1. The prime denotes the ordinary differentiation with respect to time $t\in (0,+\infty )$. We consider initial conditions $S_0=S(0)>0$, $I_0=I(0)>0$ and $R_0=R(0)=0$. In particular, the positive $I_0$ indicates the initial size of individuals who share information spontaneously when the video is exposed on the platform. We note that the total amount of people, $N(t)=S(t)+I(t)+R(t)$, in the system is increasing since $(S+I+R)'=bN(t)$. For the convenience of mathematical analysis, we will first consider the total amount of people as a constant $N=N(0)$, which leads to $S(t)+I(t)+R(t)=bNt$, and then discuss an extended model with time-varying *N*(*t*). Parameter $\beta >0$ is the rate of contact between susceptible and infected individuals. Parameter $b>0$ reflects the growth of new users in the system. Unlike the classic epidemic model where the contact always transforms susceptible into infected, the target people may directly become recovered individuals in our model. Therefore, we divide the outcomes of interactions into two parts: $\beta _1 IS/N$ and $(\beta -\beta _1)IS/N$, where $\beta >\beta _1$. Parameter $\alpha >0$ is the transition rate of infected individuals to recovered individuals. If we ignore the interactions of individuals and the effect of advertising, $I'=-\alpha I$ leads to $I(t)=I(0)e^{-\alpha t}$, which suggests that the length of the infected period is exponentially distributed with mean $\int _0^\infty e^{-ks}\text {d}s=1/\alpha$^29^. We further introduce an assumption that $b>\gamma$ leads to $S'=(b-\gamma )N>0$ when $I=0$, which reflects that advertising would not cover the whole group of new users without the promotion from active sharers.

A key feature of our model is that we consider $N^2\gamma /(N+I)$, which we will call the *advertising function* in the rest of this manuscript, to reflect the effect of advertising. This term equals $\gamma N$ when $I=0$ and monotonically decreases to 0 as the increase of *I*, which reflects that the investment of advertising is highest when there are no active shares. Moreover, there is no need for putting advertisements when the system has enough active sharers to keep transforming the susceptible individuals.

## Dynamics of the model

As we assume that $S(t)+I(t)+R(t)=bNt$, *R*(*t*) is known once we figure out the dynamics of the other two variables. Thus we reduce system (1) into a two-dimensional system

2 $$\begin{aligned} \left\{ \begin{aligned}{}&S'=-\frac{\beta IS}{N}-\frac{\gamma N^2}{I+N}+bN,\\&I'=\frac{\beta _1 IS}{N}+\frac{\gamma N^2}{I+N}-\alpha I,\\ \end{aligned} \right. \end{aligned}$$

We first illustrate the existence and uniqueness of the equilibrium.

### Theorem 3.1

If $\alpha >b$, system (2) has a unique interior equilibrium $(S^*,I^*)$ with $S^*\in (S_-,S^-)$ and $I^*\in (0,N)$, where

$$\begin{aligned} S_-=\left( b-\frac{\gamma }{2}\right) \frac{N}{\beta },\quad S^-=\left( \alpha -\frac{\gamma }{2}\right) \frac{N}{\beta _1}. \end{aligned}$$

### Proof

Solving

$$\begin{aligned} \left\{ \begin{aligned} -\frac{\beta IS}{N}-\frac{\gamma N^2}{I+N}+bN=0,\\ \frac{\beta _1 IS}{N}+\frac{\gamma N^2}{I+N}-\alpha I=0, \end{aligned} \right. \end{aligned}$$

leads to

$$\begin{aligned} \alpha I^2+\left( \alpha -\frac{\beta _1}{\beta }b\right) NI-\left( 1-\frac{\beta 1}{\beta }\right) \gamma N^2-\frac{\beta _1}{\beta }bN^2=0. \end{aligned}$$

Define

3 $$\begin{aligned} f(I):=A I^2+BI+C, \quad \text {where}\quad A=\alpha ,\ B=\left( \alpha -\frac{\beta _1}{\beta }b\right) N,\ C=-\left( 1-\frac{\beta 1}{\beta }\right) \gamma N^2-\frac{\beta _1}{\beta }bN^2. \end{aligned}$$

Since $\beta _1<\beta$ suggests $C<0$, together with $A>0$, there must exist a unique positive zero root for $f(I)=0$, denoted as $I^*$. Notice that

$$\begin{aligned} F(N)=N^2\left( 2\alpha -\frac{2\beta _1}{\beta }b-\left( 1-\frac{\beta _1}{\beta }\right) \gamma \right) > N^2\left( 2\alpha -\left( 1+\frac{\beta _1}{\beta }\right) b\right) , \end{aligned}$$

as $\gamma <b$. If we further assume $\alpha >b$, it gives $F(N)>0$ and thus suggests that $I^*=(-B+\sqrt{B^2-4AC})/(2A)\in (0,N)$.

Regarding *S* as a function of *I* based on (2) gives

4 $$\begin{aligned} \left\{ \begin{aligned} S=\frac{N}{\beta I}\left( bN-\frac{N^2\gamma }{N+I}\right) :=S_1(I),\\ S=\frac{N}{\beta _1I}\left( \alpha I-\frac{N^2\gamma }{N+I}\right) :=S_2(I). \end{aligned} \right. \end{aligned}$$

It is straightforward to tell that $S_1$ monotonically decreases from $+\infty$ to $S_-=(b-\gamma /2)N/\beta$, and $S_2$ monotonically increases from $-\infty$ to $S^+=(\alpha -\gamma /2)N/\beta _1$. Since $\beta >\beta _1$ and $\alpha >b$, we have $S_-<S^*<S^+$. This completes the proof. $\square$

### Theorem 3.2

If $\alpha >b$, $(S^*,I^*)$ is globally asymptotically stable on $\Omega =\{(S,I)\ |\ S>0, I>0\}$.

### Proof

We first analyze the local stability of the equilibrium. The Jacobian of (2) on the equilibrium is

$$\begin{aligned} J(S^*,I^*)= \left( \begin{matrix} -\frac{\beta I^*}{N} &{} -\frac{\beta S^*}{N}+\frac{N^2\gamma }{(N+I^*)^2} \\ \frac{\beta _1 I^*}{N} &{} \frac{\beta _1 S^*}{N}-\frac{N^2\gamma }{(N+I^*)^2}-\alpha \\ \end{matrix} \right) . \end{aligned}$$

Solving $|\lambda \textbf{I}-J |=0$, where $\textbf{I}$ is the identity matrix, gives

$$\begin{aligned} \lambda ^2+\bar{B}\lambda +\bar{C}=0,\quad \text {where}\quad \bar{B}=\frac{\beta I^*}{N}+\alpha +\frac{N^2\gamma }{(N+I^*)^2}-\frac{\beta _1S^*}{N}, \quad \bar{C}=\frac{(\beta -\beta _1)\gamma NI^*}{(N+I^*)^2}+\frac{\beta \alpha I^*}{N}. \end{aligned}$$

Remind that $S^*<S^-$, we have

$$\begin{aligned} \bar{B}>\frac{\beta I^*}{N}+\alpha +\frac{N^2\gamma }{(N+I^*)^2}-\alpha +\frac{\gamma }{2} =\frac{\beta I^*}{N}+\frac{N^2\gamma }{(N+I^*)^2}+\frac{\gamma }{2}>0. \end{aligned}$$

Therefore, $\bar{B}>0$ and $\bar{C}>0$ suggest that $\lambda ^2+\bar{B}\lambda +\bar{C}=0$ has two negative zero roots, which implies that $(S^*,I^*)$ is linearly stable. Furthermore, on the phase plane of (*S*, *I*), we have

$$\begin{aligned} S'=-\frac{\gamma N^2}{I+N}+bN>0, \end{aligned}$$

as $\gamma <b$, along the positive *I*-axis, and

$$\begin{aligned} I'=\frac{\gamma N}{I}>0, \end{aligned}$$

along the positive *S*-axis. This suggests that the region $\Omega =\{(S,I)\ |\ S>0,I>0\}$ is a positive invariant set. Together with the uniqueness and local stability of $(S^*,I^*)$, we obtain the global stability of $(S^*,I^*)$ . $\square$

From the above analysis, we conclude that the system (2) with any initial conditions $S_0>0$, $I_0>0$ and $E_0=0$ will always converge to the unique interior equilibrium $(S^*, I^*)$, which suggests that the number of potential audience and active sharers would converge to a fixed size as the spread of the video. From (3), it is also clear that $\beta _1/\beta$, $\alpha$, $\gamma$, *b* and *N* jointly determine the equilibrium.

**Figure 2:**
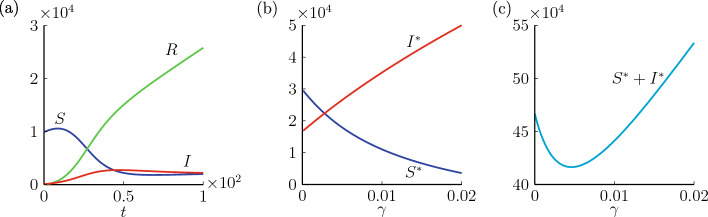
Dynamics of system (1) with $S_0=9900$, $I_0=100$, $\beta =0.4$, $\beta _1=0.1$, $\alpha =0.03$, $b=0.02$. We consider $\gamma =0.002$ in (**a**) and vary $\gamma$ from 0 to 0.02 in (**b**, **c**).

We provide numerical simulations of system (1) with initial conditions $S_0=9999$, $I_0=1$ and $R_0=0$ in Fig. 2a. Here we consider a small $I_0$ to reflect the moment of the first release of the video. To further illustrate the relationship between the advertising function and the dynamics of system (1), we vary $\gamma$ from 0 to 0.02 and show the steady states of system (1) according to (3) and (4) in Fig. 2b. As susceptible individuals have a constant growth rate, their amount undergoes an increase and then monotonically decreases when the system has enough infected individuals. We note that in our model there would not be more rebounds for the number of susceptible individuals, since the equilibrium is a stable node, and thus *S* would converge to the eigenvector of $(S^*, I^*)$. From Fig. 2b, we see that advertising affects the final size of the susceptible individuals. A larger investment in advertising would drive the susceptible individuals to maintain a relatively small size and enhance the size of active sharers. However, the total amount of susceptible and infected individuals changes with $\gamma$ in a non-monotone manner, as shown in Fig. 2c. If the goal is to keep the size of recovered individuals as large as possible, the value of $\gamma$ needs to be carefully selected.

## Evaluate advertising efficiency using agent-based algorithm

Both individual interactions and advertising contribute to the increase of recovered individuals. Since it is easy to calculate the dynamics of (2), a direct thought for evaluating the efficiency of advertising is to calculate the number of individuals who decide to watch the video after receiving advertisements. That is, one can use *S*(*t*) and *I*(*t*) to numerically calculate the integration of $S'(t)$:

5 $$\begin{aligned} \int _0^T S'(t)\text {d}t=-n_S(T)-n_A(T)+bNT,\quad \text {where }\quad n_S(T)=\int _0^T\frac{\beta IS}{N}\text {d}t, \quad n_A(T)=\int _0^T\frac{\gamma N^2}{N+I}\text {d}t, \end{aligned}$$

where $n_A(T)$ directly denotes the number of individuals leaving the class of susceptible individuals due to advertising. If we consider the initial conditions given in Fig. 2, it would lead to $n_S(T)\approx 26055$ and $n_A(T)\approx 1671$ when $T=100$. This method does not give a full landscape of the efficiency of advertising, because (5) only considers the increase of infected individuals due to advertising as the contribution of advertising. Those infected individuals recruited from advertising will transform more susceptible individuals until they become recovered. These converted individuals should also be attributed to advertising. More details of the spreading process are needed to provide an appropriate evaluation of advertising efficiency.

**Figure 3:**
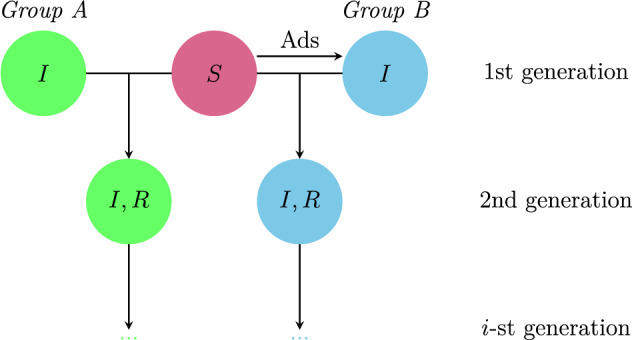
Schematic illustration of the generation of viewers.

To trace the spreading process and find out the source of infection, we now introduce the concept of ‘generation’ for viewers. The first generation of viewers are those viewers who become infected without person-to-person interactions. They come from two parts: the initially infected individuals $I_0$, which we call *Group A* and highlight in green in Fig. 3; the infected individuals converted from susceptible individuals due to advertising, which we call *Group B* and highlight in cyan in Fig. 3. When the existing infected individuals produce new infected or recovered individuals through person-to-person interactions, these new viewers become the next generation. These infected and recovered individuals in further generations can be all traced back to those viewers of the first generation. More specifically, we conclude that an individual decides to watch the video because of sharing if the corresponding first generation belongs to *Group A*. In contrast, an individual decides to watch the video because of advertising if the corresponding first generation belongs to *Group B*. However, since the differential equation model is on population-level and cannot reflect the generation relationship among individuals, other modeling approaches are needed for recording the path of infection.

Now we propose a stochastic agent-based model to mimic the spreading process. In this model, we use a Monte-Carlo realization process for some agents who change their states with time to represent the dynamics of individuals transforming through different classes. In any single realization of the stochastic model, an agent $\textbf{s}$ is either susceptible, infected or recovered. Suppose there are *O*(*t*) susceptible agents, *P*(*t*) infected agents and *Q*(*t*) recovered individuals at time *t*. We first advance the stochastic simulation from time *t* to time $t+ \tau$ by randomly selecting *P*(*t*) infected agents, one at a time, with replacement, so that any particular agent may be selected more than once, and allowing those agents to transform susceptible agents into infected or recovered. Once the *O*(*t*) potential infection events have been assessed, we then select a number of susceptible agents at random according to the value of $\gamma N^2/(O(t)+N)$ to become infected. We then add *bN* susceptible agents into the system. Next, we randomly select *O*(*t*) times of infected agents, one at a time, with replacement, allowing those agents to become recovered. Finally, we average the data from many identically-prepared realizations of the model to approximate the solution of system (1). The pseudo-code of the stochastic simulation algorithm is given in Algorithm 1. The code implemented by Julia can be found on GitHub.

**Figure:**
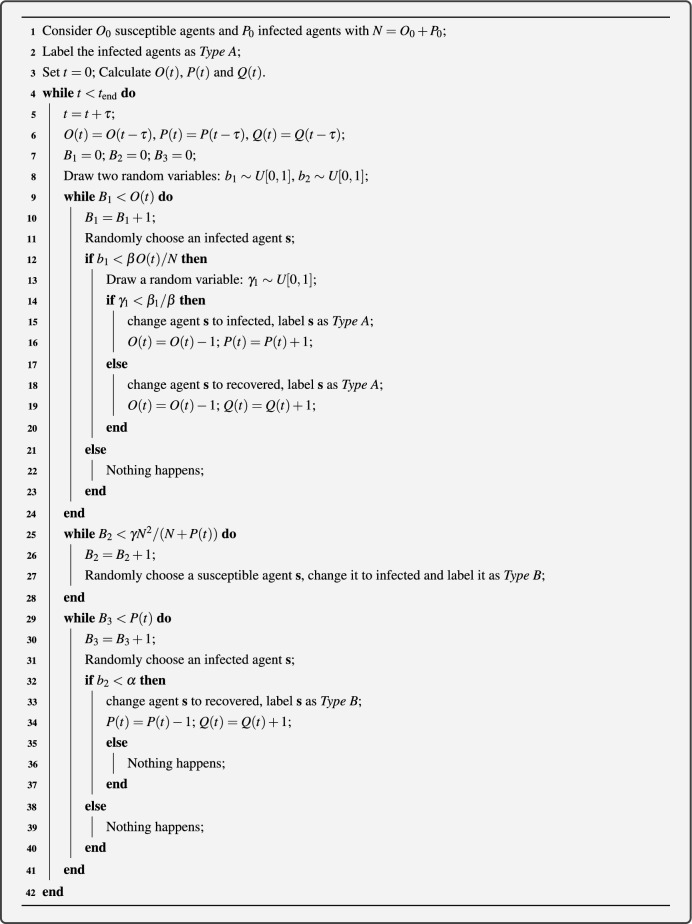
Algorithm 1 Pseudo-code for a single realization of the stochastic model

**Figure 4:**
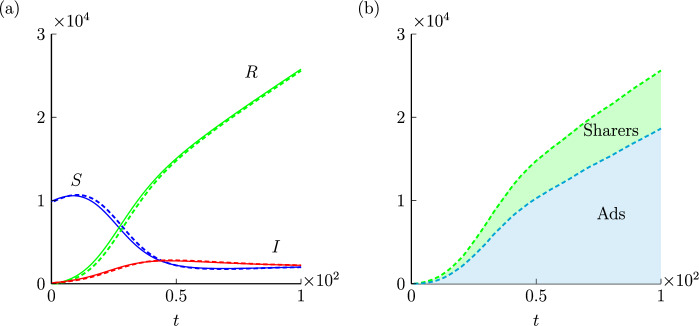
Dynamics of the agent-based model. Initial conditions are $S_0=9900$, $I_0=100$, $\beta =0.4$, $\beta _1=0.1$, $\alpha =0.03$, $b=0.02$. Dashed curves in (**a**) are the averaged number of susceptible (blue), infected (red) and recovered (green) individuals in numerical simulations. Solid curves in (**a**) are the solutions of (1). The blue region in (**b**) shows the number of individuals with *Type A*. The green region in (**b**) shows the number of individuals with *Type B*.

Numerical simulations based on the agent-based simulation algorithm are provided in Fig. 4. Fixing $\tau =1$, we perform 20 times identically-prepared simulations and average the data of *O*(*t*), *P*(*t*) and *Q*(*t*), respectively, which are drawn with dashed curves in Fig. 4a. By comparing the averaged data to the solutions of system (1), which are drawn with solid curves in Fig. 4a, we see that the solutions of the continuum model and averaged data from the agent-based model match well. The individual-level simulation results validate the population-level differential equations model. Moreover, we distinguish the viewers due to sharing, refereed as *Type A*, and the viewers due to advertising, refereed as *Type B*, and show their dynamics in Fig. 4b. It is surprising to observe that most individuals who have watched the video can be traced back to the first generation of viewers who are recruited by advertising.

## Model validation against data

Since music video is a typical kind of videos that people are interested in sharing online, we now fit our model to the viewing data of music videos from YouTube. We use the daily views data of three songs: “Caroline”, “Cheap Thrills” and “All About That Bass” collected by Sachak-Patwa et al.^20^. As the data is limited and the stochasticity in the viewing data is inevitable, instead of aiming to make perfect fits or predictions, we are more interested in seeing whether our model is capable of qualitatively describing the joint effect of sharing and advertising in the popularity of music videos.

**Figure 5:**
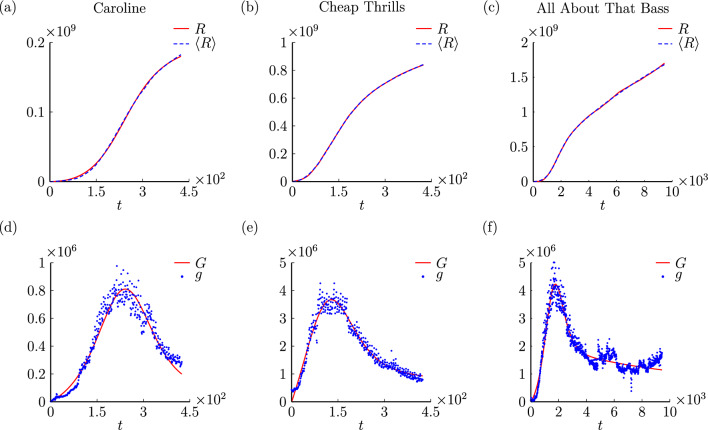
Our epidemic model (1) fitted to daily viewing data for YouTube music videos.

We consider the number of daily views on *i*th day, denoted as $g_i$, as the number of people who received the advertised information on that day. Summing daily views from 0th day to *i*th day gives the data of recovered individuals, denoted as $\left<R\right>_i=\sum _0^{i}g_i$, which associates with *R*(*t*) in system (1). We then treat $\beta$, $\beta _1$, $\alpha$ and $\gamma$ as fitting parameters, and use the ordinary least squared (OLS) method^30^ with a normalized cost function

$$\begin{aligned} \text {Er}=\frac{\sum _{i=0}^{n}[\left<R\right>_i-R(t_i,\theta _0)]^2}{(\text {max}_i{\left<R\right>_i})^2\times t_{\text {max}}}, \end{aligned}$$

where $\theta _0$ is the set of parameters and $t_{\text {max}}$ is the largest number of days. This generates an ordinary least square optimization problem

$$\begin{aligned} \hat{\theta }=\text {arg }\text {min}_{\theta \in \Omega }\text {Er}(\theta _0), \end{aligned}$$

where $\Omega$ is the feasible region for parameter values. In our model, the only requirement for parameters is that they need to be non-negative. We solve this problem by using the Nelder-Mead simplex algorithm^31^, which is a basic simplex search method. Moreover, since we do not have the data for the number of target people and active sharers, we artificially set $S_0=2\times 10^9$ according to the information on YouTube’s official blog^32^ and a small number of initial active sharers $I_0=100$.

We compare the fitted results of *R* to viewing data $\left<R\right>$ in Fig. 5a–c. Moreover, we calculate the daily views $G(i)=R(i)-R(i-1)$ where $i=1,2,3...$, and compare with the daily view data $g_i$ in Fig. 5d–f. The best fit parameters and errors for the three songs are presented in Table 1. These results suggest that our model fits well with the data. The Julia code for parameter estimation to generate these results can be found on GitHub.

**Table 1:** Best fit parameter values and the corresponding errors of the solutions as shown in Fig. 5.

Video	$\beta$	$\beta _1$	$\beta _1/\beta$	$\alpha$	$\gamma$	b	Error
Caroline	7.9561	0.3987	0.0501	0.3876	$7.5\times 10^{-8}$	$5.5\times 10^{-5}$	$4.98\times 10^{-5}$
Cheap thrills	8.9792	0.0911	0.0101	0.0870	$1.9\times 10^{-6}$	$2.0\times 10^{-4}$	$4.60\times 10^{-6}$
All about that bass	0.0302	0.0220	0.7285	0.0012	$4.4\times 10^{-4}$	$3.3\times 10^{-4}$	$1.62\times 10^{-5}$

The three groups of parameters reflect the characteristics of the spread of these songs. “Caronline” has large $\beta$ and large $\alpha$, suggesting that, on average, each active sharer can promote more individuals to listen to this song while they quickly lose the interest of sharing it. “Cheap Thrill” has a small $\beta _1/\beta$ suggesting a low rate of transformation from listeners to active sharers. “All About That Bass” has a high $\beta _1/\beta$ suggesting that there are many listeners willing to promote this song to others. A small $\alpha$ further suggests that it takes a long time for people to lose interest in this song. Moreover, a larger $\gamma$ indicates that the extensive spread of this video is accompanied by a relatively higher investment of advertising compared to the other two songs.

For “Caroline” and “Cheap Thrill”, there is a large difference in the scales between $\gamma$ and other parameters. For “Caronline”, we have $\gamma N\approx 162$. Based on the simulation algorithm of the agent-based model, there are only 162 individuals would be recruited to become active sharers in the first time step. As the increase of active sharers, the number of newly recruited active sharers in each time step would be smaller than 162. From (5) we can directly calculate that the total number of recruited active sharers is around $7\times 10^4$, which is much smaller than the size of the audience. Although we are not going to replicate the agent-based models with 2 billion agents, there is no doubt that the infection path for most audiences could be traced back to an active sharer recruited by advertising.

Simulating system (2) for six more years gives a prediction for the size of viewers, which are approximately $3.9\times 10^8$, $2.0\times 10^9$ and $3.5\times 10^9$ for “Caroline”, “Cheap Thrills” and “All About That Bass”, respectively. However, these results are higher than the real data, which are around $3.5\times 10^8$, $0.97\times 10^9$ and $2.6\times 10^9$, respectively, up to December of 2023^33–35^. This suggests that linear growth is perhaps not an appropriate assumption for the model to predict the long-term dynamics of viewing data. The time scale which our model remains valid for needs to be carefully identified. The spreading process of a video may no longer follow an epidemic manner after a fixed period of time from the date the video was originally uploaded.

## Discussion

### Modifications of the model

The assumption of constant *N* is only a proper approximation when the growth of new users is relatively low compared to the size of initial susceptible individuals. However, considering a varying *N*(*t*) would lead to a more complicated system. An autonomous system corresponding to (1) can be obtained by introducing some new variables

$$\begin{aligned} s=\frac{S}{N},\quad i=\frac{I}{N},\quad r=\frac{R}{N}. \end{aligned}$$

Substituting these variables into (1) leads to

6 $$\begin{aligned} \left\{ \begin{aligned}{}&s'=-\beta is-\frac{\gamma }{i+1}+b-bs,\\&i'=\beta _1 is+\frac{\gamma }{i+1}-\alpha i-bi,\\&r'=(\beta -\beta _1)is+\alpha i-rb, \end{aligned} \right. \end{aligned}$$

Calculating the equilibria of (6) leads to a third-order polynomial for *i*, which suggests that the dynamics of (6) could be quite complicated. Furthermore, reaching a steady state in system (6) suggests that the proportion of individuals in each class holds constant as the spread of a video, while a consistently growing number of active sharers seems not realistic for the long-term dynamics of information dissemination. We leave further modifications and analysis as future work.

## Conclusion

In summary, by investigating the dynamics of a novel epidemic model that incorporates the influence of advertising, we show that advertising plays an important role in the spread of viral videos. Similar to the spread dynamics of an epidemic, we can still rely on analyzing the properties of equilibrium to tell whether an initial state would lead to an explosive spread of information. The strength of advertising determines the final size of individuals who are not aware of the video. Through tracing the path of views for each individual based on an agent-based model, we provide the microscopic landscape of the spread process, and further distinguish the contributions from sharing and advertising. It turns out advertising remarkably facilitates the dissemination of information mostly in an indirect manner. An appropriate advertising strategy can significantly improve the spread of information, even if the investment in advertising is low.

Our modeling framework combines continuous differential equations model and discrete agent-based model. This allows us to simultaneously describe the population-level dynamics and the individual-level behaviors during the spread of information. Although in this work we focus on a simple epidemic model for highlighting the interaction of sharing and advertising, this framework could be extended to capture more complex dynamics of information spreading on social media. A promising way is to extend the mean-field ODEs model to a heterogeneous partial differential equations (PDEs) model, so that the individual-level behavioral traits can be incorporated in both continuous and agent-based models, where the heterogeneous role of individuals has received increasing attentions in studies of both sociology^36^ and epidemiology^37^. Note that technical difficulties of designing the stochastic agent-based algorithm may arise when considering the heterogeneity of traits, since the transformation between individuals with different traits may associate with complicated mechanisms such as diffusion and advection.

There are some other possible ways to extend our epidemiological modeling framework. Since we pay particular attention to the interplay between person-to-person transmission and advertising, our model ignores a variety of factors that may influence the spread of information. Incorporating realistic factors, such as the competition between different advertisements into our model may provide more profound insights into information dissemination^38^. Incorporating credibility, which is a key effect in referral marketing where recommendations from friends and families could make individuals restore their interest in the information, would lead to a system with abundant dynamics^39^. Imaginably, some kinds of circulation dynamics potentially associated with the multiple rebounds of view counts would appear^20^. For the purpose of providing more accurate prediction for the long-term dynamics of viewing data, the assumption of linear growth for the view counts of music videos could be modified with realistic mechanisms, such as time delay and forgetting effect^20,40^. Another interesting extension would be to explore the optimal control problem for viral videos, especially when a deadline is proposed for effective advertising^41,42^. We leave these potential directions for future considerations.

## Data Availability

All daily views data are presented within the manuscript (Fig. 5d–f), which were originally collected by Sachak-Patwa et al.^20^. The detailed codes used during the current study are available on https://github.com/Yifei216/ViralVideos1.git.
